# An Ecological Study of Identity in Teaching English as a Foreign Language in Light of the Dynamic Systems Model of Role Identity

**DOI:** 10.3389/fpsyg.2021.799418

**Published:** 2021-12-31

**Authors:** Rouhua Wang, Majid Elahi Shirvan, Tahereh Taherian

**Affiliations:** ^1^Changchun Institute of Technology, Changchun, China; ^2^Department of Foreign Languages, University of Bojnord, Bojnord, Iran; ^3^English Language Department, Yazd University, Yazd, Iran

**Keywords:** language teachers’ role identity, ontological beliefs, self-perceptions, Dynamic Systems Model of Role Identity, action possibilities, English as a foreign language

## Abstract

Influenced by the growing urge of investigating the combined nature of teacher identity with the dynamic teacher professional learning processes in recent years, the present study aimed to cast an ecological look at identity role construction and change in L2 teaching. To this aim, Dynamic Systems Model of Role Identity (DSMRI) meta-theoretical framework was employed with the centrality of social–cultural roles in framing an EFL teacher’s experiences and guiding actions. In a single-case study, a Chinese EFL teacher’s dynamic construction of identity (as a single-case design) was traced in three phases: before a practicum (teacher professional development program), during the practicum, and during the first year of teaching. A triangulation of data was used to ensure the adequacy and representativeness of the required data. The data were analyzed qualitatively to find traces of change and development in the teacher’s ontological beliefs, goals, self-perceptions, and action possibilities. The DSMRI-oriented analysis of pre-, mid-, and post-practicum data emphasized the traces of role identities of the teacher trainee in her professional development process, that also created both emerging patterns and emerging challenges in her role, fostering a more negotiated, adaptive and realistic teacher role identity. This study substantiated the usefulness of the DSMRI for viewing language teachers’ professional development and the dynamic identity development processes as several temporal and situated factors contribute to the alignment or misalignment of a teacher’s ontological beliefs, goals, self-perceptions, and action possibilities.

## Introduction

Identity as a personal construct is at the core of the self who is actively engaged in teaching and learning, but it resides in the periphery of academic discourse (Bochner, 1997; Stets and Burke, 2012; Eisenbach, 2016). Teacher identity, which includes a combination of self-perceptions, beliefs, values, purposes, affects, and actions at the core of teacher’s role (Bullough, 1997; Woodbury and Gess-Newsome, 2002; Beijaard et al., 2004; Sachs, 2005; Beauchamp and Thomas, 2009; Jiang and Zhang, 2021), has shown to be a major factor in guiding, elaborating, justifying, and understanding teachers’ professional lives in relation to others, society and the whole world (MacLure, 1993). Moreover, constructing an identity as a teacher is part of preserving teachers’ commitment to their job and abidance by professional norms (Hong, 2010). Teachers’ identityforms their behaviors and decisions, how they seize professional development (PD) chances, and what obligations they find inherent to their role (Beauchamp and Thomas, 2009).

Recent years have shown an emerging interest of researchers and practitioners in the complex and dynamic teacher PD processes intermingled with their identities (Beijaard et al., 2004; Geijsel and Meijers, 2005; Henry, 2016; Pennington and Richards, 2016; Barkhuizen, 2017; Kaplan and Garner, 2018; Teng, 2019; Garner and Kaplan, 2021). In this dynamic approach to teacher identity, teacher identity acts as a relevant framework for defining, exploring, and constructing teachers’ learning experiences in PD programs (Garner and Kaplan, 2018). The dynamic nature of teacher identity is continuously formed by personal, professional and contextual factors, as explored in a body of research (e.g., Pennington and Richards, 2016; Higgins and Ponte, 2017; Reeves, 2018). Some researchers have explored the contribution of personal reflections or narratives to the growth of teacher identity (e.g., Farrell, 2011; Anspala et al., 2012; Wolff and De Costa, 2017). Similarly, the role of teacher beliefs (e.g., Wu et al., 2011) and emotions (e.g., O’Connor, 2008; Song, 2017; Wolff and De Costa, 2017; Teng, 2019) in forming teacher identity has been explored.

The above-mentioned works of research share a common rejection of a static view of teacher identity. Rather, they perceive teacher identity a dynamic, changing, and context-dependent construct that can be best viewed as a constantly growing process. If we see identity as complex dynamic system with different mutually interacting dimensions (Pennycook, 2010), we cannot ignore the process, content, and structure of this system in forming or changing teachers’ action and emotions, two components of teacher identity development suggested by Kaplan and Garner (2018). But, understanding the multiple interacting aspects of identity in full seems to be challenging. One current challenge is the very diverse and often unclear treatment of teacher identity, and the challenge of concluding from the results of different methodological approaches used to explore it (Kaplan and Garner, 2018). Thus, consistent with Henry (2016, 2021), in order to explore the possibilities and consequences of a language teacher identity (henceforth LTI), we are supposed to investigate the construct from an interdisciplinary perspective.

By including other fields and investigating theoretical perspectives developed outside the applied linguistics domain, we can best reveal the strengths and weaknesses of our own domains of expertise. Particularly, in the present research, the interdisciplinary work draws on teacher education and teacher identity, areas of common interest and pedagogical proximity, and also on Kaplan and Garner’s conceptualization of LTI. We find Kaplan and Garner’s research to be especially useful for our analysis because they proposed an innovative model of teacher identity (DSMRI) which can act as a potential framework for describing identity construction of language teachers (Kaplan and Garner, 2017, 2018).

## Review of the Related Literature

In the premodern era, identity was viewed as a singular and individual entity revealed in making appraisal procedures for evaluating teachers and their development based on predetermined professional criteria (Porter et al., 2001). In the modern era, a mere focus was on teachers’ acquisition of “assets” (knowledge, efficiencies, or beliefs) as the foundation of PD. Acquisition of “assets” emphasized the significance of ideal learning outcomes in terms of “what is meant to be learnt” by teachers (Akkerman and Meijer, 2011). The premodern and modern approaches shared the presupposition of linearity in the construction of teacher identity and differences in how teachers develop throughout their profession were ignored (Beijaard et al., 2000).

As described by Richardson et al. (1998), the postmodern perception of identity was a reaction to both premodern and modern views. In this approach (from mid-twentieth century onward), identity is seen as an actively constructed entity by the individual on a continuous basis. Identity formation is dependent on several factors including inherited traditions, external exigencies, ideological constructs, and individual markers (Kumaravadivelu, 2012). From a postmodern view of language identity, researchers have valued other important factors shaping teachers’ beliefs of and behaviors in teaching which need to be integrated into teacher PD programs. Such factors form teachers’ personal perception of who they are as teachers (i.e., teacher identities) (Capps et al., 2012).

In the postmodern era, the existing literature on teacher identity has shown growing interest in the role of teachers’ educational perspective, values, perceived competencies, efficacy to do different teaching activities as well as the construction of interests, personal and social aims in the school, and mindsets about the school setting in teachers’ learning as well as decisions regarding their practice (Barkhuizen, 2017; De Costa and Norton, 2017; Reeves, 2018). Besides, researchers have acknowledged the key role of teachers’ emotions, sense of satisfaction, and frustrating experiences in their professional development (Ben Said, 2015; Benesch, 2017; Song, 2017; Wolff and De Costa, 2017; Fisher et al., 2018; Miller and Gkonou, 2018). The common theme in the existing literature is that identity is not a static feature of a teacher, but a complex, dynamic, evolving, emergent, and relational construct since teachers are faced with a multi-dimensional, continuously changing, and unstable definitions of themselves (Trent, 2014).

Following a bottom-up approach to investigating the complexity of teachers’ identity from an ecological viewpoint, Morgan and Martin (2014) invited LTI researchers to employ a classroom-as-ecosystem approach to trace the dynamics of pedagogical experiences. The ecological framework locates identity as a construct at the meso level, the association between identity and ideology at the macro level, institutional efforts at the meso level, and social activity at the micro level, which are all strongly correlated. It is the combination of macro, meso, and micro practices that eventually determines which teacher identities are legitimized in relation to language proficiency, practices, and skills (De Costa and Norton, 2017). From a complex dynamic perspective to teacher identity, Barkhuizen (2017) offered a definition of LTI to embrace many influential elements in the development of teacher identity, such as the cognitive, affective, social, ideological and historical dimensions as well as the shaky borders between an individual’s inside and outside worlds.

Reeves (2018) described teacher identity as dynamic, multi-dimensional, negotiated and co-constructed, and pointed out that neoliberal educational settings, putting external forces on curriculum and evaluation, may further cause conflicts in the dualities of external pressures and teachers’ values and beliefs. Also, based on dialogical self-theory (Akkerman and Meijer, 2011), Henry (2016) proposed a dynamic conceptualization of teacher identity, emphasizing that preservice teachers’ development can be conceptualized as multiple, relational and dynamic. Moving beyond the unraveling of developmental professional identities and their correlations, his research focused on the processes at work in the shifts among different identities.

Contemporary teacher identity literature has contributed to the acknowledgment of the dynamic nature of the construct situated in the contexts of practicum and formal settings, and has found constructs associated with many factors. However, the existing literature has heeded less to the nature of optimal formation of teacher identities, the structure and kinds of process, and outcomes in the increasingly interdisciplinary and rapidly shifting, uncertain, and complex contexts that feature the twenty-first century. Furthermore, it seems that the important role of teacher’s emotions on the process, content, and structure of language teacher identity system has been almost neglected in the existing literature. We agree with Mercer and Kostoulas (2018) argument that teachers’ emotions and their influence on teacher identity require more in-depth scrutiny.

In the present research, to analyze teacher identity, action and emotion changes, a comprehensive complex dynamic systems model (DSMRI) was used which had been developed by Kaplan and Garner (2017, 2018). DSMRI assumes teacher learning beyond change in pedagogical knowledge and skills to include change in a set of components that comprise the teacher’s identity system, such as the teacher’s purposes of teaching, self-perceived features of teaching, overall view as a teacher, perceived possibilities for action as a teacher and emotions associated with each component. More specifically, DSMRI entails assumptions about the two-way and interdependent associations among the individual’s action and affect, environmental forces, and expected behavior that fit in with the contemporary complex dynamic systems (CDS) perspective.

DSMRI represents an intuitive understanding that a teacher develops in achieving goals guided by his or her beliefs about the situation and the self as a teacher in that situation (Kaplan and Garner, 2018). In this model, the teacher’s beliefs, purposes, self-perceptions, and actions are seen as constantly developing to indicate who the individual is and how s/he acts as a teacher (i.e., the teacher’s role identity). DSMRI is marked by social psychological and sociocultural perceptions of identity (Holland et al., 1998; Stets and Burke, 2014) that comprise the main unit-of-analysis of teacher identity as the personal meaning of occupying the teacher role in a specific sociocultural situation. In DSMRI, this definition is the representation of a complex dynamic system (Opfer and Pedder, 2011; Henry, 2016, 2021)—an ever growing interactive network of role-specific assumptions and beliefs, self-conceptions, values, goals, emotions, and actions held by the individual at the core of who s/he is as a teacher in the situation (Kaplan and Garner, 2017).

At the core of DSMRI is the degree to which the individual considers being a teacher an important aspect of who s/he is. However, beyond the *level of commitment* to the teaching role, the DSMRI highlights three aspects of the teacher identity: *content*, *structure*, and *process*. Two teachers might have similar levels of commitment to their job, but have quite different assumptions, purposes, values, emotions, and perceptions about teaching (content), which might be somehow aligned with each other (structure), and grow through various processes (process). Diversity of content, structure, and process would lead to a different understanding of situations and response to events, consideration and enactment of instructional strategies, and trajectories of professional growth (Bullough, 1997). In the present research, DSMRI was employed to guide the exploration of the context-dependent development of LTI in three phases of time: before, during and after a professional development practicum. As mentioned previously, the model is compatible with the assumptions of the CDS approach and helps map the emergent patterns of LTI as constructed through the student teacher’s actual experience of professional development.

## Methodology

### Setting and Participants

This study was set in a pre-service teacher training course (a practicum) in a private language learning institute in China. This course aimed to equip student teachers with the required knowledge and practical skills of teaching English as a foreign language (EFL). It also aimed to promote their critical thinking and reflective skills as future EFL teachers. Various interactive tasks were used to let student teachers exchange ideas and practice deep thinking and reflections. In this single-case study design, the participant was a teacher trainee participating in a practicum of four weeks. The data were collected over three separate time spans with a focus on identity shifts happening (a) before the practicum, (b) during the practicum and (c) during the first year of teaching. The reason for the selection of a practicum to trace the dynamics of LTI changes is because practice learning and first year of teaching is highly challenging (Ferrier-Kerr, 2009; Trent, 2013). The first year of teaching is often marked by tensions in the construction of an integrated professional identity and prospective teachers can be uncertain regarding their profession choice (Flores and Day, 2006). In this condition, developmental identities are susceptible to change. Teacher trainees can feel tensions while holding different perceptions of teaching (Smagorinsky et al., 2004) and feel not ready to cope with the tensions of classroom experiences (Flores and Day, 2006).

The participant (henceforth named Sara) was a 23-year-old female preservice teacher participating in an EFL teacher training program. Before taking part in the program, she had spent three years of learning EFL in different private language institutes and participated in international exchange programs. These experiences enhanced her interest in English language as well. It should be noted that Sara had received high scores in almost all relevant courses, feeling engaged in experiences of higher education and chances of building her teaching skills and enhancing her English language competence. It should be mentioned that three points were considered in choosing the participant. Firstly, as the study design needed the provision of intra-personal data, it was better to select a student teacher with an amiable relation with her peers so that she could easily share her new experiences. Secondly, as a study with a CDS perspective requires detailed data gathered across the time points, it was necessary to choose a participant committed to learn best in the practicum, and who could record all her feelings, tension, conflicts, and past and present job-related experiences. Throughout the courses of the practicum, Sara had cooperated cordially with another, equally ambitious and responsible teacher trainee. Thirdly, it was required to choose a participant who, to some extent, could show uncertainties regarding her career choice, so that we can trace identity changes occasionally emerging in the data.

### Instrumentation

**FIGURE 1:**
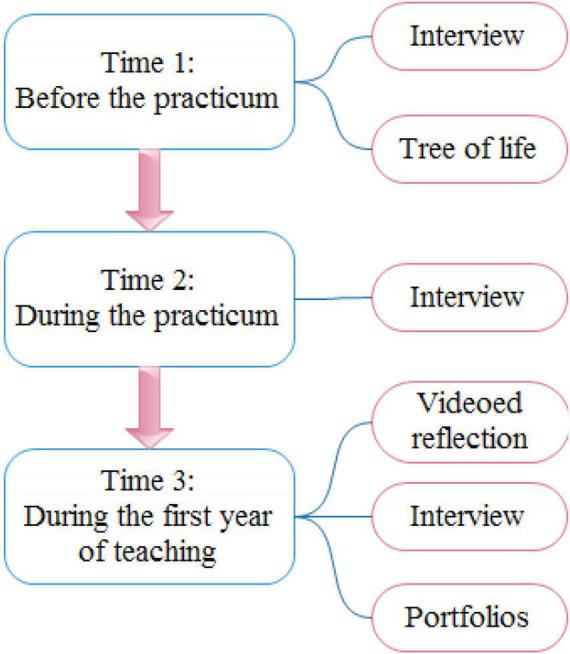
Data collection instrumentation in different phases of study.

A triangulation of data was used from multiple sources. Triangulation enhances the validity of qualitative studies by helping scholars to search for consistency among multiple repertoires of information to build themes or categories (Creswell and Creswell, 2017). Therefore, a number of instruments were used for data collection, including a regular interview with the participant, video reflection, tree of life, and portfolios (see Figure 1).

#### Tree of Life (Only Before the Practicum)

A reflective instrument used to investigate the influence of historical and cultural values is the tree of life approach (Farrell, 2007). It comes from reflective practice research and can be used as a methodological reflective tool for teachers interested in accessing their identity (Farrell, 2007). More specifically, it is a self-reflective instrument used to trace a teacher’s personal and professional development. A tree of life is separated in three parts: “roots,” “trunk,” and “limbs.” The roots represent a description of a teacher’s early effects, and as such comprise the basis of what has formed a teacher’s early years such as family values, heritage, ethnicity, religion, and socioeconomic features that have somehow shaped a teacher’s identity. At the trunk of the tree we move away from the early experiences at home to begin gaining significant experiences from early school years all the way up to a teacher’s high school years, and also focus on any experience that could have led to developing a teacher’s perspectives on teaching and teachers. The next part of reflection on the tree of life involves the limbs. The limbs represent a teacher’s experiences beyond schooling and include his or her most recent experiences and effects.

A tree of life method was used to explore Sara’s LTI before the practicum. This method managed to provide us with Sara’s self-reflections on LTI before participation in a formal course to prepare her for a real teaching job in near future. To map the tree, interviews were required to provide the content. Thus, the three parts of the tree, the root, the trunk and the limb were formed through a semi-structured interview with Sara. To construct the root, Sara was asked questions such as “To what extent did your family affect your decision to go for a teaching job?” “Did the family encourage or discourage your choice of teaching as a life employment? “Were there any ideological concerns about the teaching job you had in mind?” “Were there any socio-economic limits that could change your goals in prospective teaching job? These questions addressed the ontological roots of Sara’s conceptualization of her prospective job. Instances of questions asked to map the tree trunk are: “Which teachers did you admire most at school (or university)? What was special about them that intrigued you?” “Were the classes more student-centered or teacher-centered? In your opinion, which worked better and why?” These questions aimed to find traces of Sara’s own background as a student and impressions she received from the teachers influencing her prospective LTI. Instances of questions asked to map the limbs of the tree of life were: “Were there any other factors outside family and school to affect your perceptions of prospective role as a teacher? If any, how did they contribute to your role identity before entering the actual teaching training course and the real teaching experiences afterward?” These questions aimed to further elucidate the more inclusive context in which Sara’s LTI development was embedded.

#### Interview

A CDS design needs data to trace the evolutionary processes at both macro and micro levels, and a means of associating them together (Lichtwarck-Aschoff et al., 2008). Therefore, the time scale for conducting interviews to capture the evolutionary process as well as short and long changes were needed (van Geert and van Dijk, 2015). The interviews were held in different times: three interviews before entering the practicum, six interviews during the practicum and five interviews afterward. Also, based on the process described by Creswell and Creswell (2017), handwritten field notes were recorded during the interviews and then written again to ensure that enough information could be retrieved and documented. Meanwhile, descriptive notes were mixed with analytic notes to form a holistic view of the emerging occasions.

To explore how Sara’s LTI developed during this course which took a couple of weeks, a semi-structure interview was used, some questions of which were: “To what extent do you perceive yourself capable of acquiring the instructed teaching skills and strategies?” “To what extent do you feel free to make changes and show creativity in the instructions provided in the course?” “Are there any sociocultural factors involved in the content or structure of the instructions provided in the course?” “To what extent do you find the activity- and task-goal setting relevant and defendable?” “Is there any change to how you perceive your role as a teacher in class?” “Do you believe, your role as a language teacher should be limited to classroom learning or beyond? Please elaborate.”

#### Videoed Reflection (During the Teaching Experience)

Rather than making traditional written reflections, Sara created a video of her reflections *via* her mobile phone. Then, by reviewing her real teaching experiences, she needed to systematically reflect on the effectiveness of her teaching. In fact, videos were used as an effective instrument for supporting her personal reflections on her teaching. To facilitate the personal reflections, we provided her with a self-check list with a number of critical questions such as (a) What parts of your teaching have you found satisfactory? (b) Have you been successful in achieving your teaching objectives? (c) Do you give clear instructions? (d) What strategies do you think of to further enhance the quality of your teaching?

#### Portfolios

Sara was asked to prepare a working portfolio as a mediating instrument for her reflections on her teaching experiences and analyze critical teaching events that happened each week. Every weekly portfolio entry needed one self-selected piece of evidence which represented an insight that she learned about herself as a teacher during that week. In a four column matrix, Sara identified the entry, explained why she selected it, composed a short theme of the entry, and expressed what she had learned from it. Thus, her reflection-in-action and on-action were integrated.

### Data Analysis

**FIGURE 2:**
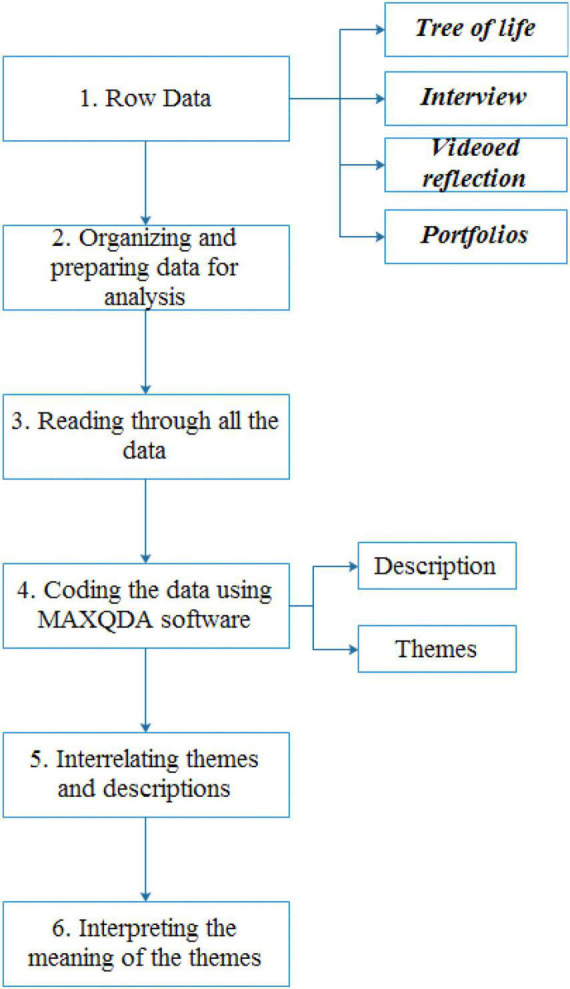
The stepwise data analysis procedure.

Creswell and Creswell (2017) suggested that researchers look at qualitative data analysis in a stepwise manner. These included movement from specific to general and in several levels of analysis (see Figure 2). Figure 2 indicates a linear, hierarchical approach building from the bottom to the top, but it is more interactive in practice; the different steps are correlated and not always followed in the order presented. We followed these steps as we started our data analysis, trying to come up with the identification of themes and descriptions, which helped us to narrow our focus down to the most important pieces of data. When all the data were collected, prior to data coding the interviews and video reflections were transcribed. Next, considering the portfolio and tree of life data as well, to find tentative codes, we carefully read the data for several times and highlighted words considered as key concepts for situations indicating properties of DSMRI. Then, we, the researchers of the study, compared these tentative codes and discussed the similarities and differences in these codes. After three rounds of discussions, they were checked for agreement, were refined, and divided into meaningful themes. Also, we explored how the teacher’s comments on her accumulated experience and competence contributed to the structure and process of her identity. This process continued until we could finalize the specification of the labels for each code. Finally, we determined the associations between the codes and categorized the themes and sub-themes. We formed a typology of different themes and descriptions in MAXQDA. These classifications were (a) self-perception, (b) beliefs, (c) purposes and goals, and (d) perceived action possibilities. For each category, we found instances of the category in action. Instances of codes included self-perception such as the teacher’s agentive behaviors in relation to her own emotions, her beliefs, references to teacher experience, and her comments on classroom interpersonal relationships, among others. Eventually, in the second phase of data analysis, we shifted our focus to the structures and processes of identity formation.

## Results and Discussion

In what follows the results are presented based on different changes at three points of time: before the practicum, during the practicum, and after the practicum during the first year of teaching. Then the effect of the cultural context of the prevailing configurations on the DSMRI will be reported.

### Phase 1 (LTI Before the Practicum)

Analysis of Sara’s overall accounts showed that in the pre-practicum phase, influenced by her family and the society, she was to think of a limited range of jobs for her future. Her goal-setting in life was, thus, not wholly intrinsically motivated. Her family, influenced by the traditional society, perceived a teaching profession appropriate for ladies. However, the subject (EFL teaching) was wholeheartedly to her own interest as she found the EFL classes “as a source of going beyond limitations and finding a way into a whole new world,” in her account. Thus, evidently, she acknowledged the liberalizing and promising nature of the English language studies.

### Possible Future Self

At school, Sara was inspired by a few teachers, whom she described as “strict yet very knowledgeable and understanding.” As reflected in her tree of life data, she mostly experienced teacher-centered education, which she described as “typical of her school days marked by teacher-fronted classes and a lot of students together, accommodated within one small room.” In such a context, underachievement was mostly attributed to “student laziness,” a very common term used in Chinese schools, and success to both the student’s hard work and teacher’s outstanding teaching qualities (these seemingly concern the ontological and epistemological beliefs component of DSMRI), also raised by Weiner (2011). Sara’s accounts revealed that her perception of her prospective teaching role (the self-perception and definition component) and the professional goals she was to set for near future (the purpose and goals component of the model) were significantly influenced by the social, ideological, and cultural settings before entering the practicum. Below is an extract from her elaborations:

*I was seriously thinking about the teaching job for my near future. There were not many other choices I had. Becoming a teacher would represent the most appropriate image of me in family and society. There is no resistance to this job for women in my country and that means a lot (interview).*

This final point could also be linked to the action possibilities component of DSMRI (Kaplan et al., 2015), pointing out how the perceived existing possibilities can limit action. This finding also shows how two or more variables or components (e.g., goals, self-perceptions and action possibilities) within an identity-construction model can interact to mark the development of a construct of interest (Kaplan et al., 2015; Garner and Kaplan, 2018). Similar works of research indicative of these interactive effects include Bandura (1997), Dweck (2006) and Zimmerman (2008).

### Phase 2 (LTI During the Practicum)

Sara entered the preservice practicum to prepare for real teaching experiences soon afterward. The practicum included 80 hours of teacher training in a well-credited private language institute with many branches throughout the country.

### Shifts in Sara’s LTI

The analysis of Sara’s answers revealed, firstly, a significant change from her perceived teacher-fronted role (typical of her own experience of school days in phase 1) to a learner-centered teaching role. This can be observed in the following excerpt:

*Here in the training course, we are constantly advised to seat students in a circle and keep walking in class so that the attention is equally distributed among students. We are also advised to keep teacher talking time to minimum and instead increase the chances for student talks. I am not expected to convey knowledge anymore. The trainer keeps reminding us that we are not responsible for student learning. We can at best be only responsible for our own teaching (interview).*

There seems to be a radical shift from Sara’s perceived role of teacher as a container of knowledge to teacher as a facilitator or negotiator of knowledge. This is related to the ontological/epistemological beliefs component of the DSMRI. Sara viewed this changing role somehow as a function of the subject to be taught (English language) and the environment of teaching (i.e., the private sector). Below is a relevant account:

*The whole expected way of teaching English seems to be different from schools. At schools, as I remember, the teachers were supposed to teach only some fixed material through a fixed way of teaching, without even caring about the real proficiency level of students in class. But, here, in the private sector, as I am being instructed how to teach in my classes, there is more and more emphasis that we are flexible and should weigh up conditions all the time before we decide what is best to be taught and how (interview).*

The above-mentioned account has another evidence for a shift of LTI from the pre-practicum to the during the practicum phase. The change appears to be ontological/epistemological in type. It can be also related to goals/purposes, as in the public school experience of teaching, a teacher pursues more personal goals (i.e., to do her pre-assigned job as already planned and scheduled). But, in the private sector, the teacher is expected to set more context-dependent goals. This is also a piece of evidence for the impact of the educational context (public vs. private) on the perceived LTI.

### Contributing Factors to Sara’s LTI

In her response to the question addressing the other contributing variables to her perception of LTI, Sara drew her attention to the financial resources available to the private language institutes (including her institute of affiliation) and the schools she experienced as student life from childhood until her application for an EFL teacher’s position. Compare the following excerpt from her elaborations:

*Here, facilities are much more. Expectations are higher too. But I think it is worth it. They will provide us (in real classes) with all technologies we need, audiovisual aids and so on. The building is well-equipped and we have many teaching aids and realia we might need for teaching. We have a wider choice for sure, and can teach the same content in quite many ways, each useful in one way or another. However, when I remember my school days, teachers had to stick to the book, and even had no choice but to skip the listening or writing tasks because we either had no proper audiovisual aid or no sufficient time (interview).*

The issue raised in this account can be discussed in relation to the perceived action possibilities component of the DSMRI. Presumably, Sara’s LTI is changing during the practicum as she admits that the social and educational context can tremendously affect a teacher’ perceived actions possible to take. She begins to see a wider range of actions, which can empower a teacher to act selectively and creatively to meet the immediate situational needs. Sarah’s perception of a rather stable, uncreative and predictable teacher role seems to be developed into a dynamic, creative and empowered teacher role. Moreover, she is learning that a teacher-centered role identity is no longer cherished and needs to be replaced by a communicative, learner-centered role identity.

### Phase 3 (After the Practicum)

In this phase, Sara experienced her real FL teaching in the institute. The data were collected in her first year of service, through interviews, portfolios and videoed reflections. Sara noted very prominent fluctuations in her role identity components within the first year of her teaching. These fluctuations highlighted her continuous identity exploration, which included continuous learning, external and internal negotiation of ontological beliefs about the nature of learners and the institute, reconsideration and redefinition of goals for her students and for herself, and finally reflection and exploration of self-perceptions and action possibilities triggered by events she experienced as involving negative emotions and identity tensions.

### Misalignment Between Goals and Action Possibilities

In her first experience of teaching, Sara found some misalignment between two dimensions of her identity system (i.e., goals and action possibilities). Her goal for students was to prepare them to write an essay in a cooperative context in which students and the teacher work with each other in the process of writing an essay. However, some confusion arose during her first class in that she could not follow her lesson plan. Below is an example of Sara’s identity exploration during her first experience of teaching English to teenagers with low level of proficiency and motivation:

*During my first experience of teaching, I was placed with lowest performing 9–12 year- old English learners in a crowded class (about 18 students). In one of my first lesson plan I wrote for students I was quiet ambitious. I intended to do a joint writing construction with them, have them read their own essays, and finally have them give each other feedback. However, writing up argument and evidence out of them took up the whole class. I couldn’t understand why they had such issues. It was a disaster and I was disappointed (portfolio).*

As indicated in this excerpt, before coming to class, Sara had some positive emotions regarding her first experience. She had a high level of motivation by saying “I was quiet ambitious.” However, she could not follow her lesson plan indicating that “writing up argument and evidence out of them took up the whole class,” “it was a disaster,” and also saying “I couldn’t understand why they had such issues.” In this state, Sara had a negative feeling toward herself. In fact, an ongoing event that mismatched to her general expectations and goals led to this negative emotional episode. The mismatch between Sarah’s goal, perceived possible actions and the tension that she experienced was used as reference to judge where she was as compared to where she wanted to be. By talking to the supervisor of the institute, she could analyze the problem in her class. After reflection on her teaching, she changed her ontological belief about her teaching style. She put forth it as below:

*I thought I had planned my lesson carefully, but after I had talked about the problem to the supervisor everything made sense to me. He told me it was great success to have some of these students here in the English class. I realized that my students and I were from different worlds. I used to be a highly motivated student and tried to seize every chance to learn English. However, most of my students had a low level of motivation. Most of them were forced to learn English because of their parents’ expectations. I found out that I had demanded too much from my students but scarcely supported them (videoed reflection).*

### Alignment Between Ontological Beliefs and Action Possibilities

Also, new tensions occurred between identity elements which shook Sara’s role identity structure and elicited experiences of ambiguity and stress. This managed to trigger Sara’s reflection on her ontological beliefs (student role) that generated a new action possibility (i.e., demand less and support more) that aligned to her goals. That resulted in the reconsideration and redefinition of goals for her students and action possibilities triggered by the nature of the classroom. Compare the following excerpt from Sara’s accounts:

*As soon as I started reflecting on my teaching style and how I could meet my students’ needs, they began to enjoy more and respond more. I adapted my teaching style to their needs. It did not take long before I saw improvement in my students’ learning as well as engagement in class. This experience really changed my beliefs that it is vital that I as a language teacher should deeply know my students and how they learn specially when they come from a different language background from mine; I should adjust my teaching to their specific needs (interview).*

As seen in this excerpt, the role of the context of classroom is salient. In fact, it could be concluded that the cultural configuration (the students’ low level of motivation and Sarah’s negotiation of the problem with the supervisor) provides, at first, a guide to her role identity formation by the activation of the epistemological belief in teaching methodology and, as a result, this emerged belief causes a change in her action. Therefore, in line with Kaplan and Garner (2018), it could be concluded that inconsistencies in teacher role identities formation across different socio-cultural context indicates the relative salience of attractors in that given context. Similarly, in Ruohotie-Lyhty’s (2018) exploration of EFL teaching emotions and professional competence, misalignment between the original identity and the setting brought about a significant emotional load in the teacher and resulted in her ambiguity regarding her professional competence. By the same token, for a newly qualified foreign language teacher, the wider range of educational responsibilities of almost all teachers was not consistent with her initial idea of being a subject teacher. Moreover, feeling pressured to accept a general educational role was dissatisfying for her.

### Alignment Between Ontological Beliefs and Goals

In Sara’s development of LTI in the third phase of the study, certain alignments with the model were also found. For one, much alignment in teacher role identity manifested when Sara explained about her high engagement in implementing her goals (i.e., improving students’ skills in spelling and punctuation during the writing an essay) that she perceived as a very appropriate and effective action to pursue her goal in line with her beliefs about the situation. This led to the coherence in her role identities and positive feelings and a sense of satisfaction:

*Although I adapted my teaching to suit my students, I certainly did not change my expectations and goals. However, it caused another challenge as my students were not happy. I remember one student said “Oh miss, you were my favorite teacher until now.” I replied: “I don’t care if you hate me over this. You need to understand how important correct spelling and punctuation are.” I then explained if they apply for any university, they will be judged based on their writing in the application letter. Figuratively, it was a battle at first and convincing my students of the importance of writing skill was so challenging but gradually my students improved their writing skills. I felt satisfied and proud. This proved that students need challenging goals and that with the right teaching strategies and support their needs will be met (portfolio).*

The above-mentioned account showed Sara’s identity negotiation of goals, epistemological beliefs, and action possibilities mediated by students’ needs of learning the writing skill. This kind of identity exploration was facilitated by the practicum program, which emphasized that there should be a negotiation among teacher and students about their goals of the course and their needs. Sara’s sense of satisfaction and coherence can be considered a tool for conscious or unconscious judgment while monitoring her effectiveness of epistemological beliefs and perceived success in attaining goals. This is in line with what Schutz et al. (2018) claimed. They believed the type and intensity of emotions depend on the teacher’s appraisal of how satisfactory her pursuit of goal is within a classroom setting. Such evaluations of the success in classroom management are also associated with attributions with regard to the justifications for success and failure.

### Emotional Reactions to Misalignment Between Beliefs, Goals, and Action Possibilities

Respectively, in our data, Sara reported how judgment of experiences regarding her incoming beliefs, goals, and action possibilities induced emotionally-suggestive reactions that might either question, or confirm how she perceived herself as a teacher. For instance, Sara was deeply involved in teaching an activity that did not go on as planned. See below:

*Any teaching activity that didn’t go as planned sent me on a downward spiral of self-criticism and insecurity. So, this is what I do every day after teaching a lesson: beating my head against the wall. Sometimes I find it hard to be happy about my performance even though I receive a positive feedback from others. There were times when I felt mad that I was unable to reach the level that I wanted (portfolio).*

### Emotional Reactions to Alignment Between Beliefs, Goals, and Action Possibilities

When her first year of pre-service teaching ended, Sara referred to her changes in her pedagogical beliefs due to her engagement in understanding how she could manage her negative emotions. By the end of her one year actual experience of teaching in class, a positive change in her emotion regulation occurred as described below:

*I try to celebrate small things now. I welcome feedback from my supervisor and do my best to work in areas which needed improvement. Eventually, I realized that I was being too hard on myself. I aim to gain more and more experience and learn from mistakes. No one can be perfect. That means expecting too much of yourself. I will never stop growing and wishing to learn more. I cherish my positive and negative feelings as they are signs of my weaknesses and strengths (portfolio).*

The above-mentioned account has signs of identity negotiation which, as defined by Ruohotie-Lyhty (2018), is a process in which teachers construct their identities to better adapt themselves to the contextual requirements and to build their professional development. In these situations, teachers feel confident to maintain their original thoughts of being a teacher but they can express salient fluctuations in the way they view themselves as professionals (Ruohotie-Lyhty, 2013; Ruohotie-Lyhty and Moate, 2016). Teacher professionalism in such cases is strengthened *via* situational development opportunities.

Overall, it appears that throughout the three phases of study, Sara experienced a dynamic journey of LTI development. This dynamicity was influenced by environmental needs as well as individual needs and expectations. The sociocultural context (and its haphazard changes) in which her entire developmental journey was embedded affected her identity exploration and negotiation. Through time she learned how to overcome her negative emotions and develop a more professional and adaptive identity. She moved from a teacher-centered teaching style to a more student-centered style. Also, she learned how to overcome her perfectionistic teacher role identity and replace it with a more realistic one. The changes she experienced in LTI confirmed the effects of social context, subject domain, personal dispositions and culture (especially in the first two phases of study) on the four components of DSMRI (i.e., epistemological/ontological beliefs, purposes and goals, self-perceptions, and perceived action possibilities). The practicum managed to show her a whole different view of the EFL teaching context, which challenged her old perceptions of a teacher role in class (and beyond class). This initial shock was then soothed by her enthusiasm to experience a more negotiated role identity. Her teaching experience was a chance for identity exploration while experiencing a broad range of matched and mismatched primary contextually constructed and interdependent components that underlie teacher action. Throughout the whole experience (traced in the present three-phase study), she developed a dynamic LTI marked by an interaction of the underlying context-bound components of teacher action, as indicated by the dynamic model of role identity by Kaplan and Garner (2017, 2018) and also ratified in a body of research already reviewed (e.g., Henry, 2016, 2021; Barkhuizen, 2017; Reeves, 2018).

## Conclusion

The present study described a conceptualization of role-identity and professional development as a complex dynamic system and its use in L2 teachers’ professional role-identity. DSMRI conceptualizes role identity as a dynamic system along four components that are constantly emerging to affect motivated action through procedures that meet complexity assumptions: mutual dependence of components, non-reductionism, non-linearity, non-determinism, and context-dependence. This study showed how DSMRI could be used in a case study analysis of the role identity and professional change of an EFL teacher before taking part in a practicum (a teacher training course), during the practicum and through her first year of teaching experience. The findings substantiated the dynamic and negotiated nature of professional identity development. The study also proved the effective use of the model to inform theory building, research, interventions, and evaluations in teacher’s role identity and professional development that describe these phenomena as complex dynamic systems.

## Data Availability Statement

The raw data supporting the conclusions of this article will be made available by the authors, without undue reservation.

## Ethics Statement

Ethical review and approval was not required for the study on human participants in accordance with the local legislation and institutional requirements. Written informed consent for participation was not required for this study in accordance with the national legislation and the institutional requirements.

## Author Contributions

All authors contributed to the development of the manuscript including the data collection, data analysis, and the writing phase.

## Conflict of Interest

The authors declare that the research was conducted in the absence of any commercial or financial relationships that could be construed as a potential conflict of interest.

## Publisher’s Note

All claims expressed in this article are solely those of the authors and do not necessarily represent those of their affiliated organizations, or those of the publisher, the editors and the reviewers. Any product that may be evaluated in this article, or claim that may be made by its manufacturer, is not guaranteed or endorsed by the publisher.
